# Evolving fracture management: the role of helical plating in orthopaedic trauma surgery – a narrative review

**DOI:** 10.1007/s00068-025-02871-1

**Published:** 2025-05-12

**Authors:** Moritz Kraus, Boyko Gueorguiev, Tatjana Pastor, Ivan Zderic, Mark Lenz, Matthias Knobe, Frank J. P. Beeres, R. Geoff Richards, Hans-Christoph Pape, Torsten Pastor

**Affiliations:** 1 AO Research Institute Davos, Davos, Switzerland; 2 Department of Trauma, University Hospital Zurich, University of Zurich, Zurich, Switzerland; 3 Department of Plastic and Hand Surgery, Inselspital University Hospital Bern, University of Bern, Bern, Switzerland; 4 Department of Trauma, Hand and Reconstructive Surgery, Jena University Hospital, Friedrich Schiller University Jena, Jena, Germany; 5 Department of Orthopaedic and Trauma Surgery, Westmuensterland Hospital, Ahaus, Germany; 6 Medical Faculty, University of Zurich, Zurich, Switzerland; 7 Medical Faculty, RWTH University Aachen, Aachen, Germany; 8 Department of Orthopedic and Trauma Surgery, Cantonal Hospital Lucerne, Lucerne, 6002 Switzerland

**Keywords:** Helical plating, Long bone fractures, Biomechanics, Nerve damage prevention, Humeral shaft fractures, Distal femoral shaft fractures

## Abstract

**Purpose:**

This narrative review systematically compiles and analyzes existing literature on the use of helical plates in orthopaedic trauma surgery. By synthesizing data across various study types, it provides a comprehensive overview of the biomechanical characteristics, clinical outcomes, and anatomical advantages of helical plating.

**Methods:**

A systematic search was performed using PubMed and Web of Science databases, employing defined search terms to identify relevant studies. Single case reports were excluded, while structured case series were included. Retrieved studies were categorized into five groups: simulation studies, biomechanical studies, case series, clinical comparative studies, and anatomical studies.

**Results:**

The review identified studies from 1992 to 2023, with most of the research focusing on the femur (7 studies) and humerus (6 studies). Biomechanical studies (7) were the most common, followed by clinical case series (7), comparative studies (4), and finite element analyses (3). European institutions contributed to the majority of research, with additional studies from Asia and South America. No randomized controlled trials were found. Helical plates demonstrated comparable stability to straight plates, with distinct biomechanical advantages: superior torsional resistance in femoral fractures and improved neurovascular safety in humeral fractures.

**Conclusion:**

Helical plates offer a viable alternative to straight plates in long bone fractures, particularly for protecting neurovascular structures. Optimal designs vary by location, with 45° helical plates recommended for humeral minimally invasive plate osteosynthesis, 180° helical plates for young patients with femoral fractures, and 90° helical plates in geriatric double plating constructs. Further high-quality research is needed to establish definitive clinical guidelines.

## Introduction

The aim of this narrative review is to consolidate the existing clinical, anatomical and biomechanical knowledge surrounding helical plating—a fixation technique mainly used for treatment of proximal humeral shaft and distal femoral shaft fractures. These fractures represent a significant portion of long bone fractures and pose substantial challenges in orthopaedic trauma practice. Humeral shaft fractures represent approximately 5% of all fractures [1]. Femoral shaft fractures account for 5% of all femoral fractures with an incidence of 40 per 100,000 [2, 3]. While surgical intervention via open reduction and internal fixation (ORIF) is widely recognized as the standard treatment for suitable candidates [4], the intricacies of selecting an optimal surgical approach are multifaceted, requiring a careful evaluation of patient-specific factors.

The distribution of these fractures is bimodal, predominantly affecting younger individuals through high-energy trauma and older adults via low-energy incidents [1]. In geriatric patients, the inability to comply with weight-bearing restrictions necessitates a treatment focus on achieving maximum stability to enable early mobilization [5] leading to shorter hospital stays and better patient outcomes [6]. Conversely, in younger patients, where adherence to weight-bearing limitations is feasible, treatment strategies are often complicated by the presence of concomitant injuries, leading to increased risks of infection and delayed bone healing [7].

Straight plates have traditionally been the cornerstone for fracture fixation in both these anatomical regions. However, their use in the femoral and humeral region has raised concerns about potential risks to vital anatomical structures such as the radial nerve and the medial neurovascular structures of the thigh [8–10]. Helical plates, introduced by Fernández [11], offer a promising solution to this challenge with their design that wraps around the bone.

To delineate, a twisted plate, first presented in 1992 [12], represents a planar plate subjected to axial torsion. Torque is the cause, twist is the effect, and torsion is the internal response. Expounding on geometrical definitions [13], a spiral is defined as a planar curve emanating from a central point and progressively distancing from this origin. In contrast, a helix is defined as a three-dimensional curve generated by a point’s rotational movement around a linear axis, accompanied by a parallel displacement [11]. This definition is pivotal in understanding the true architectural essence of helical plates. Notably, a twisted plate can be considered a specific instance of a helical plate with a radius of *r* = 0, while a straight plate is akin to a helical plate with an infinite pitch.

**Fig. 1:**
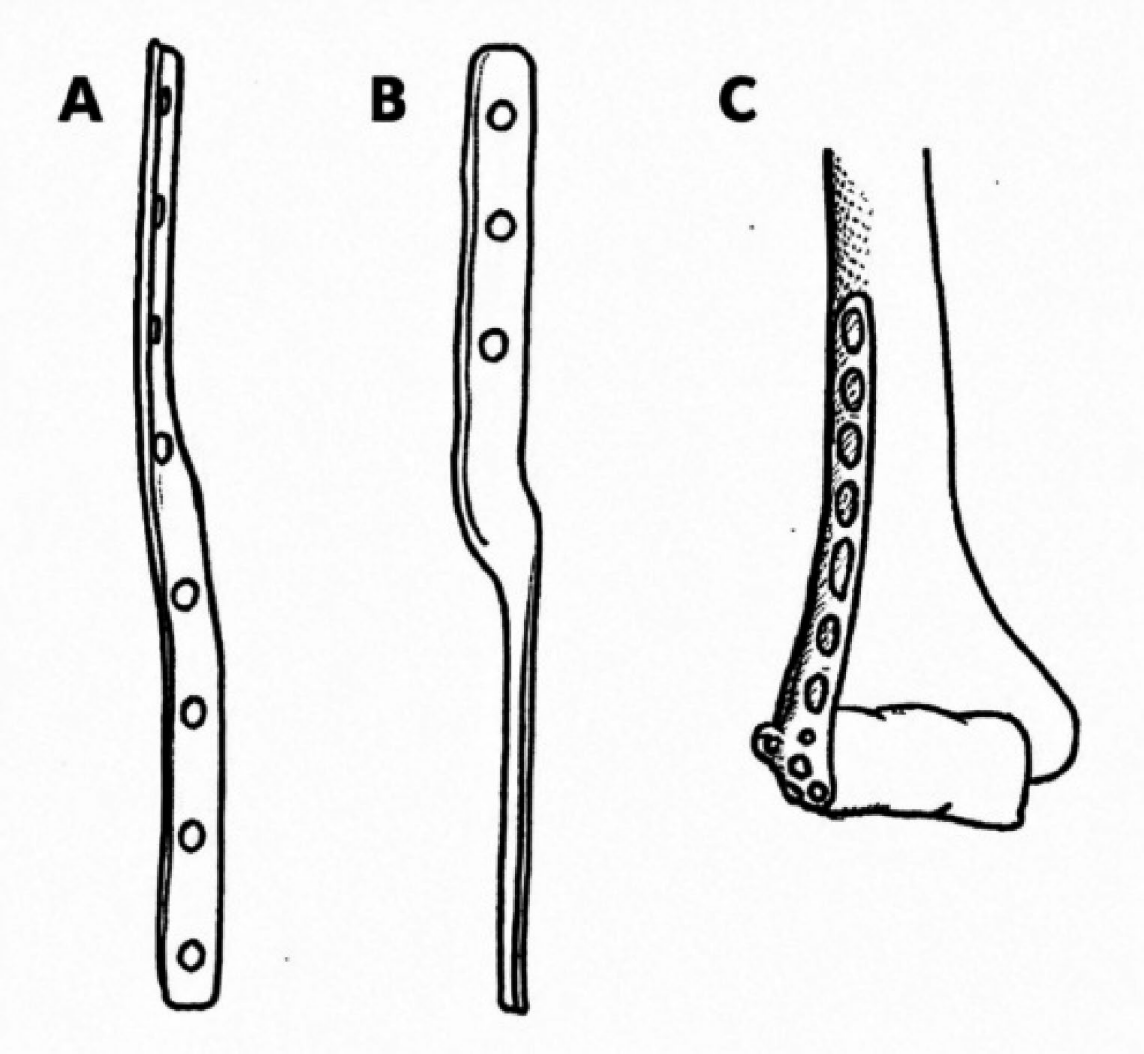
Outline of two twisted plate designs as proposed by Kumar [12], (**A + B**) and a modern plate design (**C**). **A**: Twisted plate for humeral shaft fracture fixation. **B**: Twisted plate for tibial shaft fracture fixation. **C**: Johnson&Johnson MedTech 3.5 LCP lateral distal humerus plate with a small lateral arm for perpendicular screw fixation

The historical context provides further clarity. Kumar’s study marked the initial application of twisted plates, demonstrating efficacy across multiple skeletal sites (Fig. 1) [12]. Nowadays some modern plates (e.g. Medartis Aptus Tri-lock clavicle plates or Johnson&Johnson MedTech 3.5 LCP lateral distal humerus plate) might be considered as variants of this historic design as they offer additional arms or bended holes in order to allow screw placement perpendicular to the screw angulation in the plate.

Fernández’s introduction of the first helical plate represented a significant evolution in this domain [11], with applications noted in various anatomical regions [11]. Helical plates are further applied by its inventor as reinforcement in delayed or nonunion cases additionally to a straight plate [14].

Despite the potential advantages of the helical plates, the literature to date presents limited consensus on their optimal design and application [15]. The implications of altering straight plates into helical configurations intraoperatively, particularly regarding their fatigue resistance and long-term durability, demand further investigation [16].

The aim of this review study is to systematically evaluate and compare the biomechanical efficacy, anatomical advantages, clinical outcomes, and specific challenges of helical plating versus traditional straight plating in the treatment of humeral and femoral shaft fractures.

While the central emphasis lies on helical plates, it is imperative to acknowledge twisted plates, as they represent the foundational predecessors of helical plates. This historical perspective is not merely of academic interest but is crucial for understanding the evolution of the implant design and for inspiring the development of future orthopaedic technologies. Additionally, the review addresses the practical aspects of intraoperative plate bending. It is recognized that manually contoured plates during surgery will seldom achieve a perfect helical structure and instead will incorporate elements of twisting. This practical reality underscores the importance of understanding the interplay between helical and twisted plate concepts, as it bears significant implications for both the design of new implants and the optimization of surgical techniques in fracture fixation.

To the best of our knowledge, this narrative review represents the first of its kind to systematically compile and analyze the existing body of literature on the use of helical plates in orthopaedic trauma surgery. It synthesizes data across a variety of study types, providing a unique and comprehensive overview of the field to date.

## Methods

### Search strategy

A narrative review framework was implemented, aggregating and analyzing a broad spectrum of research, crucial for elucidating the comprehensive implications of helical plating technologies in surgical practices. It was underpinned by a systematic and thorough search protocol, utilizing the extensive resources of PubMed and Web of Science databases. A set of carefully chosen search terms was employed: “(helical[All Fields] OR twisted[All Fields]) AND (‘bone plates‘[MeSH Terms] OR (‘bone‘[All Fields] AND ‘plates‘[All Fields]) OR ‘bone plates‘[All Fields] OR ‘plate‘[All Fields])”. Single case reports were excluded and structured case series were included. The literature search was completed on 31.01.2024.

The search strategy was tailored to encompass a wide range of studies, capturing the diverse dimensions of helical plating research. The retrieved articles were categorized in five distinct groups: simulation studies, biomechanical studies, case series, clinical comparative studies, and anatomical studies.

## Results

### Search results

The analysis of the dataset pertaining to helical and twisted plating yielded a range of studies conducted over a period extending from 1992 to 2023. Of the 32 studies that came up in the first search, 23 were included into this narrative review after full-text screening. A total of seven studies focused on the femur making it the most frequently investigated bone. The humerus was subject of six studies. Other anatomical regions of interest included the clavicle, tibia, and ulna, each being represented in a single study, while one study specifically examined sheep tibia.

In terms of study types, clinical case series were the most common investigations, with a total of six studies conducted. Biomechanical investigations were identified in seven studies, where two of the seven were conducted using artificial bones. Clinical retrospective comparative studies were noted in four instances. Finite element simulations were explored in three studies, anatomical analyses were performed in five studies, and a single study provided an overview of the topic.

Geographically, the distribution of studies was concentrated in Europe and Asia. European institutions, specifically from Switzerland, Austria, Germany, Spain, Italy, and Turkey, contributed to a total of thirteen studies. In contrast, Asian research, emanating from countries such as South Korea, India, China, and Singapore, was represented in five studies. Additionally, there was one study from South America.

No randomized controlled trials were detected, indicating a potential area for future research. This gap in the literature underscores the need for future research, particularly high-quality empirical studies, to determine the efficacy and application scope of helical plating more conclusively in orthopaedic trauma surgery.

### Simulation studies

Finite element analysis (FEA) has provided new perspectives on the biomechanical efficiency of helical plating systems in the field of fracture therapy. A study comparing the deformation and stability of straight versus helical compression plates in transverse and oblique fractures on sheep tibiae concluded that helical plates demonstrated superior fracture gap closures and torsional resistance under axial compression loads compared to straight plates [17]. Here it is vital to take into account that helical plates cause more shear and rotational movement under axial stress in horizontal fracture gaps compared to straight plates [18]. The merging of computational and experimental approaches in this work demonstrated that helical designs excel in stabilizing transverse fractures.

Zhang et al. [19] looked at the biomechanical features of Herbert screws and helical plate fixations in midshaft displaced clavicle fractures. While Herbert screw fixation mirrored the stress distribution of an unbroken clavicle and was appropriate for minor fractures, helical plate fixation achieved more stability. The former was related to stress shielding, indicating a preference for helical plate fixation in patients requiring early return to activity with restrictions on postoperative shoulder mobility and weight-bearing.

Further investigation into the hemi-helical plate (HHP) demonstrated its improved ability for oblique fracture repair, which is particularly effective in helical cracks caused by torsional stresses and comminuted fractures [20]. The circumferential HHP design provides compressive strength at fracture sites and exceeds straight plates in terms of fracture-holding capability and flexibility under varying loading situations, so it enhances both axial and torsional stiffness in synthetic bone constructs, offering effective load distribution [16]. Both experimental data and FEA supported this. Furthermore, Krishna et al. [20] found higher resistance to screw pullout as compared to straight plates and reasoned this due to the different screw angulation in helical plate constructs.

A recent FEA study [21] reveals that helical plating leads to slightly deferred bone healing due to larger shear movements, offers clinical benefits like reduced stiffness, lower neurovascular risks, and improved load distribution.

These findings add considerably to the orthopaedic knowledge by showing the enhanced stability provided by helical plating systems in certain fracture configurations. They open the path for future fracture fixation technology advances and possible better results for complicated fractures. However, higher torsional and shear movements at the fracture gap were detected in FEA studies, possibly influencing the fracture gap healing [21].

### Clinical studies and comparative analyses

#### Case series on twisted plates

Three clinical case series on helical plating have been published so far, one of them dealing with humeral fractures, one—with femoral fractures, and one—with both and additional proximal tibial fractures.

In their case series, Kumar et al. [12] introduced a 90° twisted plate for orthopaedic fixation, applying it to 24 humeral, 6 tibial, and 2 radial fractures with varying hole counts: 8 for humerus, 6 for tibia, and 4 for radius. The novel plate’s strength was compared to that of a standard flat plate. The twisted plate was found to be 49% stronger against bending and 132% stronger against twisting (Fig. 1).

Bülhoff and colleagues explored a 95° twisted plate to enhance the stability of subtrochanteric fracture fixation in combination with intramedullary nailing, although the number of cases in this study was not specified [22]. More recently, Nicolacai et al. [23] reported on 24 cases using Zimmer Biomet’s anatomic locking plate system (ALPS)—a 45° twisted plate with an additional anterior kink to avoid the deltoid insertion during humeral fracture fixation—, achieving a 100% union rate with only a single instance of iatrogenic temporary radial palsy.

#### Case series on helical plates

Four clinical case series on helical plating have been published so far, all of them dealing with humeral fractures.

Along with describing his technique on helical plating, Fernández presented 20 cases involving multifragmentary proximal humeral fractures, fixated with manually pre-contured helical plates [24]. The patients were treated using periosteal implants positioned laterally at the proximal humerus and anteriorly at the distal humerus. The cases included nonunion fractures extending to the proximal part of the bone and comminuted humeral shaft fractures categorized as 3- and 4-part proximal humeral fractures. However, the clinical outcome of this patients has not been reported.

Yang [25] described 9 cases of comminuted humeral fractures treated with manually pre-contoured helical plates angled at 90° to spare the deltoid insertion and additional bone grafting in two cases, finding that all fractures healed within 14 to 28 weeks without significant complications, though there were instances of hardware removal and two unsatisfactory outcomes. Moon et al. [26] analyzed the treatment of 12 humeral fractures using manually pre-contoured plates based on a cadaveric humerus model, utilizing a 90° helical angle. Their approach included 5 limited-contact dynamic compression plates (LC-DCP) with 12 holes and 7 long PHILOS plates with 10 holes, noting one case of delayed fracture union. Garcia-Virto et al. [27] reported on 15 AO/OTA 12 C humeral fractures treated with a 90° contoured helical plate, featuring at least 4 distal locking screws, with a mean Constant Score of 72 ± 13 points after 6 months.

A primary issue identified in case reports is the lack of consensus on the optimal design of the osteosynthesis plates, compounded by the challenge of standardizing intraoperative contouring across various surgeons. Nevertheless, it is evident that for Minimally Invasive Plate Osteosynthesis (MIPO) of the humerus, a 45° helical configuration is most suitable as it is pushed through the weaker middle part of the deltoid insertion, which is not possible with 90° helical plates and the ALPS [28, 29]. In ORIF procedures, a higher degree of angulation, up to 90° or 45° with an additional anterior kink like in the ALPS plate, may be beneficial for better positioning of the plate anterior to the deltoid muscle attachment. Nevertheless, this approach is not feasible with MIPO techniques as it could potentially cause more harm and long-term weakening of the strong anterior portion of the deltoid muscle [28].

### Comparative studies

Comparative studies have exclusively investigated the outcomes of humeral fractures, providing focused insights into the effectiveness of different treatment approaches.

A retrospective study comparing helical plating to straight PHILOS plating for shoulder function after one year revealed comparable outcomes in 30 patients. Both treatments yielded good shoulder function and there were no significant differences in normalized Constant Scores or surgical complications between the two groups [30].

A decade-long study compared straight versus helical PHILOS plates in treating humeral shaft fractures in 62 patients [9]. No iatrogenic radial nerve damage was reported in the helical plate group, while two cases of nerve damage occurred in the straight plate group, though being not statistically significant. The findings suggest that helical plating might safely prevent radial nerve damage.

Comparing the efficacy of pre-contoured plates shaped on artificial bones (Synbone, Zizers, Switzerland) versus 3D-printed models for proximal third humeral shaft fractures, a study by Wang et al. [31] found that 3D printing significantly reduced both the surgery duration and blood loss. The two groups had similar outcomes regarding fracture healing and functional scores, indicating 3D printing’s value in simplifying the surgical procedure.

Comparing the lateral approach with the use of straight plates versus MIPO approach with 45° and 90° helical plates for treatment of humeral shaft fractures [32], the study found that while the operation time was shorter for the lateral approach, the overall complication rate was significantly lower for MIPO with helical plates. Both methods achieved satisfactory results, but the helical plates demonstrated advantages in complication rates [33].

The comparative studies on humeral fractures reveal that helical plating is as effective as traditional straight plating in terms of functional recovery and safety, with additional benefits such as possible lower radial nerve damage and fewer complications, while 3D printing technology in plate contouring significantly reduces surgery time and blood loss, arguing for a wider application of these techniques.

### Biomechanical characteristics of helical plating

Five biomechanical investigations have been reported in the literature so far, two in the humeral shaft region and three in the femoral shaft region, all dealing with custom-bent helical implants and none with the ALPS.

In the humeral region, straight plates, intramedullary nails, 45° helical plates and 90° helical plates have been compared in an artificial bone model using a non-destructive quasistatic test setup [29]. It was concluded that 90° helical plates were associated with higher fracture gap movements in the sagittal plane (flexion / extension) Nevertheless, they demonstrated improved resistance against displacements in the coronal plane (varus / valgus) compared to straight plates during pure bending. In contrast, 45° helical plates demonstrated equitable biomechanical competence as straight plates. The authors considered 45° helical plates as valid alternative to straight plates from a biomechanical perspective. However, no cyclic tests were performed, and only artificial bones were used. Furthermore, all investigated plate designs revealed less resistance to axial deformation under axial loading as compared to intramedullary nails. Nevertheless, all plates demonstrated higher resistance to torsional loading which was due to nail toggling [29]. The second biomechanical study compared 90° helical plates with straight plates in a human cadaveric bone model under torsional cyclic loading [18]. The authors concluded that 90° helical plating is associated with lower resistance to flexion/extension and internal rotation with bigger shear interfragmentary displacements as compared to straight plating and therefore cannot be considered as its real alternative [18].

Given the upper extremity’s major exposure to torsional stress and the prevalent method of contouring plates by torsion, such contoured plates may perform suboptimal [34]. This might be the reason why 90° helical plates performed inferiorly compared to straight plates when loaded under cyclic torsion. On the other hand, 45° helical plates seem to be a true alternative to straight plates in the upper extremity from a biomechanical perspective, however, biomechanical research under cyclic loading in a human cadaveric bone model is still pending to confirm these results.

Double plating in orthopaedic surgery stabilizes fractures using two bone plates, optimizing load distribution, reducing stiffness-related complications, and enhancing stability compared to single-plate systems [35]. In the femoral region one study compared 180° helical plating to conventional straight lateral plating in an artificial bone model simulating a distal femoral fracture in young patients [35]. The authors concluded that helical plating showed higher shear and flexion movements. Yet, it demonstrated improved initial axial stability and resistance against varus/valgus deformation compared to straight lateral plating. Besides that, the helical plating was associated with significantly higher endurance to failure and may be considered as valid alternative to lateral straight plating [35].

### Double plating definition

The second study evaluated double plate constructs in geriatric distal femoral fractures using a paired cadaveric bone model under cyclic axial loading [36]. One group was instrumented with two straight plates while the other group was instrumented with a medial 90° helical plate. The authors concluded that for geriatric patients, helical double plating with an additional 90° medial helical plate presents a biomechanical advantage over straight double plating by offering better damping during axial loading and avoiding the medial neurovascular structures, addressing the issue of excessive stiffness associated with straight plates [36].

The third study evaluated a similar double plate construct with an additional medial 90° helical plate and found an increased axial and torsional construct stiffness. The authors recommended that its use should be considered in very demanding situations for gap fractures, where single plate osteosynthesis provides inadequate stiffness for fracture healing and induces nonunion [16], as it has been shown that double-plate osteosynthesis or use of one intramedullary and one extramedullary implant improves stability [37, 38].

These five studies reveal an insight into the biomechanical behavior of the different helical plate designs under various loading conditions. The 180° helical plate behaves like a spring when axially loaded—like in the lower extremity—which leads to better resistance to failure, however, it comes with greater shear and torsional stresses at the fracture gap. The 90° helical implant in a double plate construct reveals similar improved damping capabilities under axial load, however, the additional lateral straight plate compensated for the torsional and shear forces at the fracture gap.

## Discussion

### Anatomical advantages through helical plates in the humerus

**Fig. 2:**
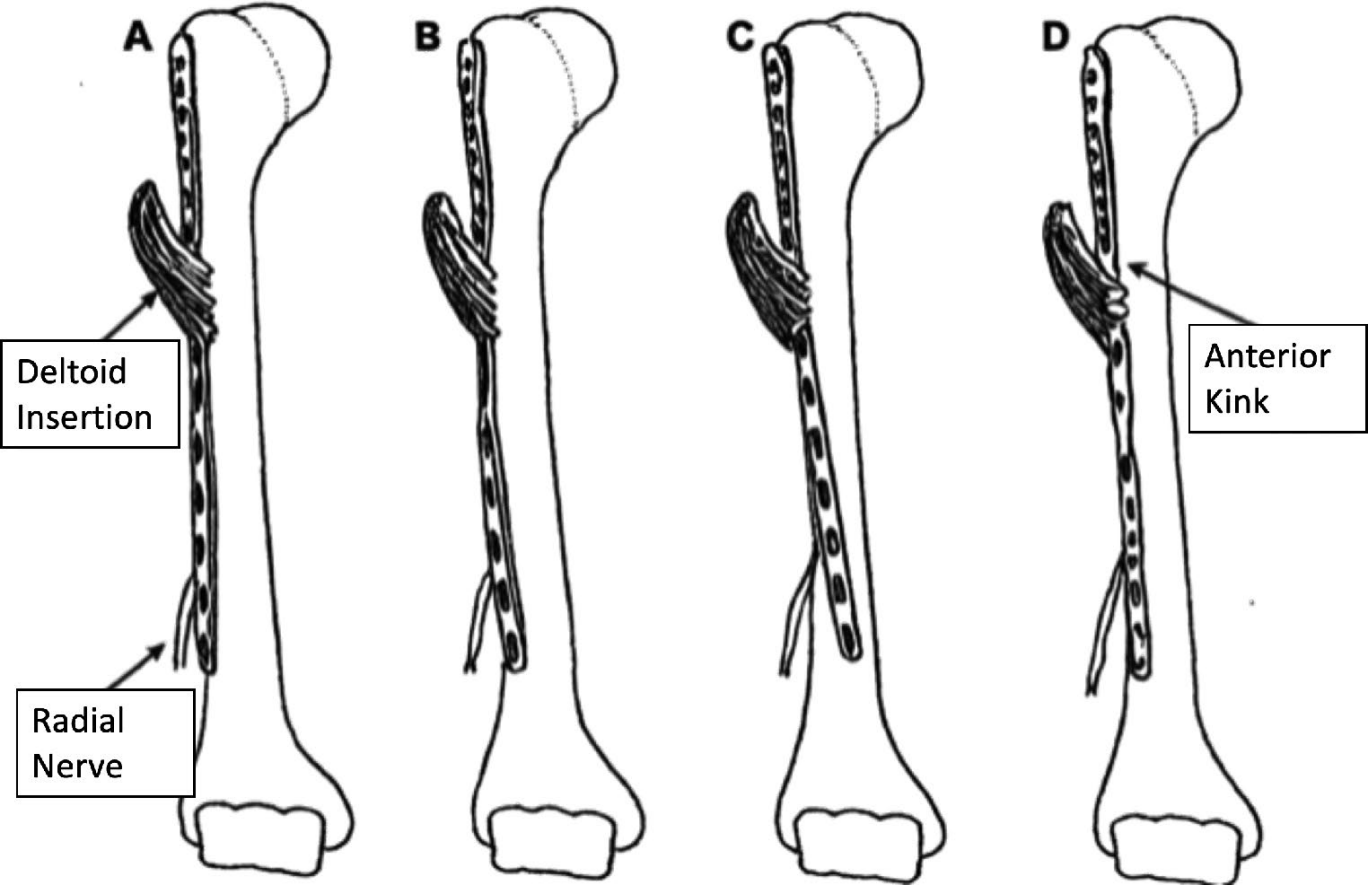
Outline of different helical plate designs and their relation to the deltoid insertion and the radial nerve **A**: straight lateral standard configuration of long PHILOS plate. Note the close anatomic relationship to the radial nerve. **B**: 45° helical contoured long PHILOS plate. Note how the straight plate and 45° helical plate are pushed through the weak middle part of the deltoid insertion and how the 45° helical plate avoids the radial nerve. **C**: 90°helical contoured long PHILOS plate. Note the violated strong anterior part of the deltoid insertion. **D**: 45° twisted Anatomic Locking Plate System (ALPS). Note the anterior kink to avoid the deltoid insertion and the 45° twist to avoid the radial nerve

Helical plating in the management of humeral fractures represents a significant advancement in minimizing nerve damage, especially concerning the radial and axillary nerve (Fig. 2). Traditional ORIF methods pose risks to the radial, axillary, and musculocutaneous nerves, with the radial nerve being particularly susceptible to palsy either from the initial trauma or after surgery [39]. This condition manifests as sensory loss in the first web space of the hand and impaired wrist and finger extension [40]. The radial nerve’s consistent anatomical pathway, as detailed by Artico et al., traverses approximately 121 ± 13 mm from the lateral humeral epicondyle to the posterior aspect of the humerus—a crucial factor in surgical planning [41].

A cadaveric study on humeral fractures demonstrated the anatomical advantage of pre-contoured helical PHILOS plates, which demonstrated a significantly reduced plate-bone distance, implying decreased axillary nerve elongation and a lower risk of nerve damage during plate insertion with MIPO technique through a delta split approach, thus being a significant consideration in humeral fracture management [42].

The design of the helical plate specifically targets these concerns. It strategically covers the lateral side of the proximal third of the humerus, avoiding the biceps’ long head, and extends to the anterior side of the middle/distal third, circumventing the radial nerve and deltoid insertion during ORIF with 90° helical plates and ALPS [11]. This configuration significantly diminishes the likelihood of radial nerve palsy. Additionally, Klepps et al. highlighted the potential damage to the anterior deltoid if more than a fifth of its insertion is released during the procedure [43], a risk mitigated by the 45° helical plate’s design, which passes through the weaker middle part of the deltoid’s insertion. In contrast, the 90° helical plate and the ALPS plate affect the strong anterior part of the deltoid insertion when inserted with MIPO technique [28].

An additional study focusing on proximal humeral fractures discussed the feasibility of helical plating through a less invasive approach, pinpointing the musculocutaneous nerve as the primary structure at risk during percutaneous screw placement. The identification of a consistent ‘danger zone’ for the nerve location in relation to the greater tuberosity underscores the importance of anatomical knowledge in surgical planning to ensure the safety of minimally invasive procedures [39].

The placement of bicortical screws in helical plating is critical. These screws, vital for anatomical reduction, must avoid the radial nerve’s most hazardous zone, identified as between 47% and 53% of the humeral length measured from the lateral epicondyle [44]. Even with these precautions, iatrogenic radial nerve palsy can occur.

There is currently no agreement on the best helical or twisted design for humeral plates. However, from an anatomical point of view, 45° helical plating is more suitable for MIPO as the implant can be pushed through the weaker middle part of the deltoid insertion. During ORIF the deltoid insertion can be completely spared with 90° helical plates and ALPS. Anatomically and topographically, all helical designs outperform straight humeral plates in terms of save distances to the radial nerve, however, the musculocutaneous nerve and the brachial artery must be considered during their application as well [28].

### Anatomical advantages of helical plating in the distal femur

Distal femoral fractures (DFF) might be challenging in orthopaedic trauma surgery, having a bimodal distribution of low-energy fractures in the elderly and high-energy trauma in younger individuals, and occurring at a rate of 8.7 per 100,000 annually [45]. Treatment complexities in the elderly include managing periprosthetic fractures and osteoporotic bone, whereas in younger patients DFFs are often associated with soft tissue or vascular injuries. It is therefore important to distinguish between these two patient populations.

**Fig. 3:**
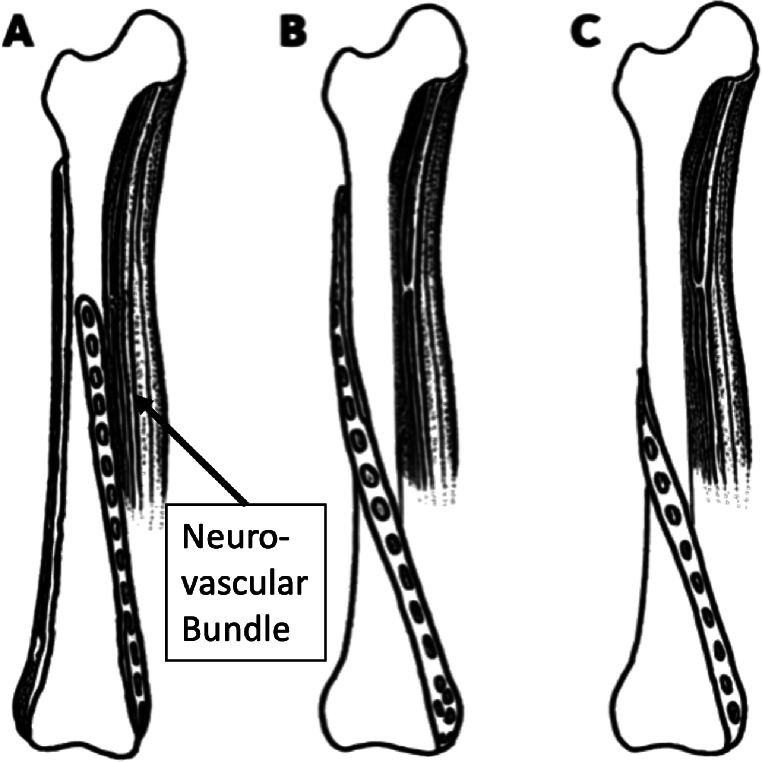
Outline of different helical plate designs and their relation to the neurovascular bundle of the thigh. **A**: straight lateral standard distal femur plate with a 90° medial helical plate in a double plate construct used for geriatric patients. **B**: 180° single helical plate. **C**: 55° helical plate as described by Hohenberger et al. [53]

The standard treatment of DFF in young patients is intramedullary nailing, especially when the knee joint is not involved [46]. Plate osteosynthesis is preferred in cases with narrow medullary canals [47], distal shaft fractures involving the joint [48], or additional intraarticular or vascular injuries [49]. The lateral approach for minimally invasive plating of the distal femur is common, providing direct bone access [50]. Yet, complication rates can vary widely [51]. Issues like iliotibial band irritation from lateral plating often necessitate hardware removal [52], while a straight medial plate can endanger the thigh’s medial neurovascular structures [53]. MIPO with a medial approach is a safe technique for the distal third of the femur, observing significant distances between the plate and critical neurovascular structures, thereby mitigating the risk of anatomical damage [53]. However, longer straight plates inserted from distal-medial endanger the medial neurovascular structures of the thigh (Fig. 3).

In this context, the use of a 180° helical implant, extending from the distal-medial to the proximal-lateral aspect of the femur, emerges as a promising solution as it avoids these critical anatomical structures [15, 24]. Hohenberger et al. performed a similar study with a single 55° helical plate and found it safe and feasible for MIPO in the distal femur [53]. Within the realm of distal femoral orthopaedic interventions, particularly helical plating via MIPO, an understanding of the femoral artery’s anatomy is crucial. The artery, branching from the deep femoral artery below the inguinal ligament, divides into several perforating vessels behind the femur towards the vastus lateralis compartment [54].

The insertion of a 180° or a 55° helical plate from a medial window in the distal femur requires careful attention to the proximal lateral thigh’s perforating vessels. The proximal-lateral MIPO approach, involving ligation of these vessels before plate insertion, aims to prevent complications like undetected bleeding [50, 55, 56].

In geriatric patients with distal femoral fractures, the treatment goal is consequently to achieve maximal stability and allow for early mobilization. Therefore, double plating with two straight plates is one treatment option. However, it endangers the medial neurovascular structures of the thigh when an additional long straight plate is used medially [10]. An alternative is an additional medial 90° helical plate from distal-medial to proximal-anterior which avoids these structures as recently demonstrated [15]. The spatial relationship between the helical plates and the femoral artery, especially at the vastoadductor membrane, has been shown as adequate for safe MIPO with both 90° and 180° plate designs as well as with 55° helical plates [15, 53]. The observed closest mean distances—particularly for longer 90° and 180° helical implants—necessitate precision not only in screw placement but also during initial implant insertion, given their proximity to the femoral artery [56]. Additionally, the proximity of the descending genicular artery’s (DGA) osteoarticular branch to the distal end of the plate highlights the risks associated with medial approaches to the distal femur [56, 57], and the variability in DGA branching patterns further complicates surgical planning [58].

### Areas for further research and investigation

Advancing fracture management necessitates prioritizing research on the biomechanical stability and durability of helical plating, particularly in osteoporotic bone and under various loading conditions [59], while advanced computational models like FEA could explore the interaction between plate design and bone healing dynamics [22]. Clinical trials focusing on patient outcomes are essential to establish the efficacy of helical plating, thereby informing about optimal use cases and enhancing patient care.

Furthermore, biomechanical research evaluating the characteristics of the ALPS is not yet available. The evolution of helical plates from manually bent straight plates raises questions about material property alterations impacting implant stiffness and strength. The pre-contouring on 3D-printed bone models [60] or direct manufacturing of plates in a helical form could potentially optimize these properties. A critical research area is comparing the material properties of 3D-printed helical implants with those of milled counterparts—a question with profound implications for the future of patient-specific implant development [61]. The development of advancements in biocompatible materials and intelligent implants capable of tracking the healing process represents key areas for innovation [62].

### Limitations

Despite the promising findings, our review acknowledges significant gaps in the literature, particularly the absence of randomized controlled trials and a consensus on the optimal design, which is exacerbated by the heterogeneity in surgeon-led contouring approaches. This gap indicates a scope for future research to refine the application and efficacy of helical plating. Scopus was not included in this review.

## Conclusion

Helical plates offer a viable alternative to straight plates in long bone fractures, particularly for protecting neurovascular structures. Optimal designs vary by location, with 45° helical plates recommended for humeral minimally invasive plate osteosynthesis, 180° helical plates for young patients with femoral fractures, and 90° helical plates in geriatric double plating constructs. Further high-quality research is needed to establish definitive clinical guidelines.

## Data Availability

No datasets were generated or analysed during the current study.
